# From idiopathic pulmonary hemosiderosis to ENaC channelopathy: a novel *SCNN1G* mutation

**DOI:** 10.1093/ajrccm/aamag320

**Published:** 2026-07-07

**Authors:** Qi Peng, Zaixue Jiang, Yongyi Chen, Yufeng Li, Fangfang Zhang, Lijun Yang, Wei Lai, Xiaojun Huang, Linsheng Wang, Xiaomei Zeng, Baimao Zhong, Xiaomei Lu

**Affiliations:** Laboratory Department, Dongguan Eighth People’s Hospital (Dongguan Children’s Hospital), Dongguan, Guangdong, China; Department of Medical and Molecular Genetics, Dongguan Institute of Pediatrics, Dongguan, Guangdong, China; Key Laboratory for Children’s Genetics and Infectious Diseases of Dongguan, Dongguan, Guangdong, China; Department of Medical and Molecular Genetics, Dongguan Institute of Pediatrics, Dongguan, Guangdong, China; Key Laboratory for Children’s Genetics and Infectious Diseases of Dongguan, Dongguan, Guangdong, China; Department of Pulmonary and Critical Care Medicine, Dongguan Eighth People’s Hospital (Dongguan Children’s Hospital), Dongguan, Guangdong, China; Department of Pulmonary and Critical Care Medicine, Dongguan Eighth People’s Hospital (Dongguan Children’s Hospital), Dongguan, Guangdong, China; Department of Medical and Molecular Genetics, Dongguan Institute of Pediatrics, Dongguan, Guangdong, China; Key Laboratory for Children’s Genetics and Infectious Diseases of Dongguan, Dongguan, Guangdong, China; Department of Pathology, Dongguan Eighth People’s Hospital (Dongguan Children’s Hospital), Dongguan, Guangdong, China; Department of Radiology, Dongguan Eighth People’s Hospital (Dongguan Children’s Hospital), Dongguan, Guangdong, China; Department of Pathology, Dongguan Eighth People’s Hospital (Dongguan Children’s Hospital), Dongguan, Guangdong, China; Department of Medical and Molecular Genetics, Dongguan Institute of Pediatrics, Dongguan, Guangdong, China; Key Laboratory for Children’s Genetics and Infectious Diseases of Dongguan, Dongguan, Guangdong, China; Laboratory Department, Dongguan Eighth People’s Hospital (Dongguan Children’s Hospital), Dongguan, Guangdong, China; Department of Medical and Molecular Genetics, Dongguan Institute of Pediatrics, Dongguan, Guangdong, China; Key Laboratory for Children’s Genetics and Infectious Diseases of Dongguan, Dongguan, Guangdong, China; Department of Medical and Molecular Genetics, Dongguan Institute of Pediatrics, Dongguan, Guangdong, China; Key Laboratory for Children’s Genetics and Infectious Diseases of Dongguan, Dongguan, Guangdong, China; Laboratory Department, Dongguan Eighth People’s Hospital (Dongguan Children’s Hospital), Dongguan, Guangdong, China; Department of Medical and Molecular Genetics, Dongguan Institute of Pediatrics, Dongguan, Guangdong, China; Key Laboratory for Children’s Genetics and Infectious Diseases of Dongguan, Dongguan, Guangdong, China

A 24-year-old woman presented with a 7-year history of recurrent hemoptysis, showed no response to immunosuppressants, and was initially misdiagnosed with idiopathic pulmonary hemosiderosis (IPH) based on hemosiderin-laden macrophages (Figure 1A, B). High-resolution computed tomography revealed acute alveolar hemorrhage without bronchiectasis (Figure 1C). Secondary causes, including autoimmune and cardiovascular etiologies, were excluded (Table S1).

**Figure 1:**
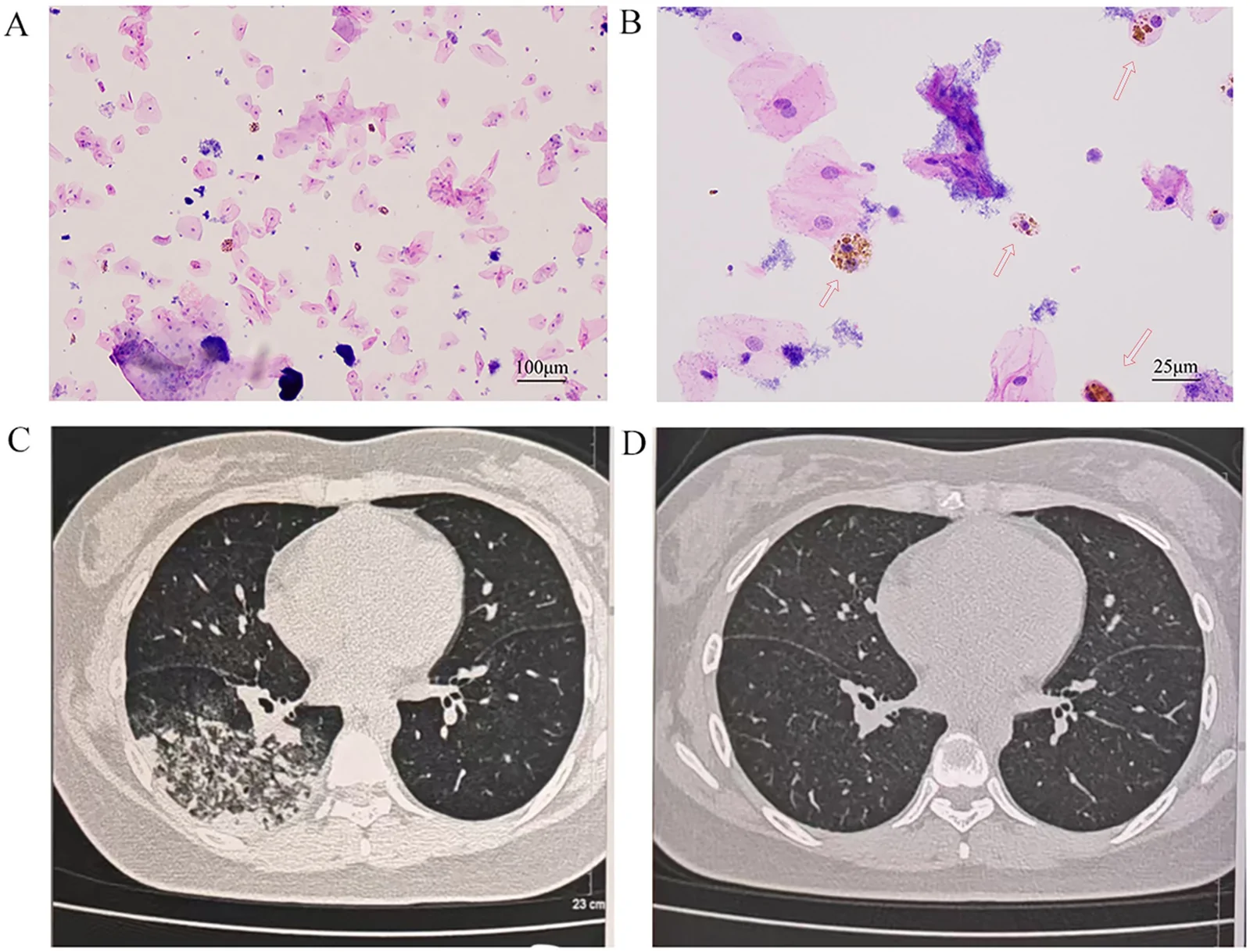
Cytological and radiological findings of the patient. (A) Low-magnification (100×) hematoxylin and eosin staining of the patient’s bronchoalveolar lavage fluid demonstrating numerous hemosiderin-laden macrophages scattered throughout the field. Scale bar = 100 μm. (B) High-magnification (400×) image showing characteristic intracytoplasmic golden-brown hemosiderin granules (arrows). Scale bar = 25 μm. (C) High-resolution computed tomography (HRCT) at presentation, demonstrating diffuse bilateral ground-glass opacities and focal consolidation in the right lower lobe, indicating acute alveolar hemorrhage. (D) Follow-up HRCT after airway-directed therapy, showing significant resolution of the pulmonary lesions and infiltrates.

Whole-exome sequencing identified a novel heterozygous variant (c.1126G > A; p.Gly376Arg) in *SCNN1G,* which encodes the γ subunit of the epithelial sodium channel (ENaC) (Figure 2A), and this was confirmed by Sanger sequencing (Figure 2B). Following confirmation of mutant γ-ENaC expression (Figure 2C) and complex assembly via α/β-subunit co-transfection (Figure 2D), patch-clamp electrophysiology revealed a gain-of-function phenotype with amplified amiloride-sensitive sodium currents (Figure 2E).

**Figure 2:**
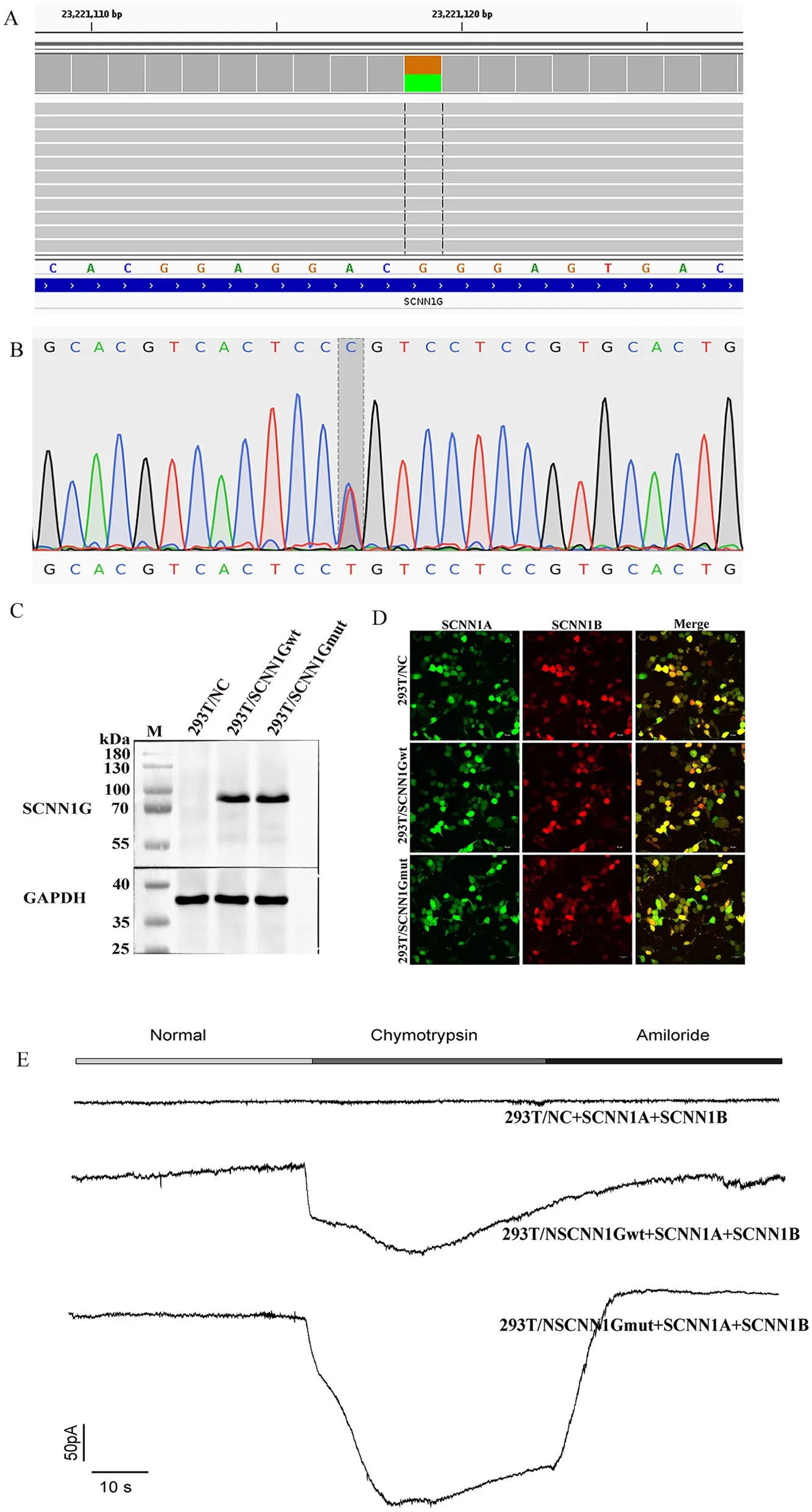
Genetic and functional validation of the *SCNN1G* c.1126G > A (p.Gly376Arg) variant. (A) Integrative Genomics Viewer snapshot of whole-exome sequencing data, identifying a heterozygous missense mutation (c.1126G > A, p.Gly376Arg) in the *SCNN1G* gene (NM_001039.4). (B) Sanger sequencing chromatogram confirming the heterozygous c.1126G > A variant (shaded gray box). (C). Western blot analysis of HEK293T cell lines (293T/NC, 293T/*SCNN1G* wild-type [wt], and 293T/*SCNN1G* mutant [mut]). Both wild-type and mutant γ-epithelial sodium channel (ENaC) proteins showed robust and comparable expression at approximately 75 kDa, with GAPDH as the loading control. (D) Immunofluorescence microscopy confirming the co-expression and proper membrane localization of α-ENaC (green) and β-ENaC (red) subunits in the γ-ENaC established cell lines. (E) Representative whole-cell patch-clamp electrophysiology traces. In cells co-expressing α/β subunits with mutant γ-ENaC (G376R), the amiloride-sensitive sodium current (activated by chymotrypsin and inhibited by amiloride) was significantly increased compared to the wild-type group, confirming a gain-of-function effect.

ENaC regulates airway surface liquid volume and mucociliary clearance.^1^ We hypothesize a novel channelopathy wherein hyperactive ENaC might drive fluid absorption and mucociliary impairment.^2^^,^^3^ Though requiring further validation, the ensuing chronic inflammation could injure the alveolar-capillary membrane, leading to recurrent alveolar hemorrhage.

Following the initiation of an exploratory regimen aimed at this mechanism—inhaled hypertonic saline (3%) targeting airway hydration, alongside oral eucalyptol and azithromycin intended to inhibit ENaC^4^—we observed a reduction in hemoptysis frequency from 41% to 17.2% over 6 months, coinciding with radiographic resolution (Figure 1D).

This case proposes a potential link between ENaC gain-of-function and alveolar hemorrhage, suggesting genetic evaluation for refractory IPH. While a temporal clinical improvement was noted, in vivo models remain essential to confirm if ENaC hyperactivity directly mediates capillary injury. Furthermore, ENaC’s role in alveolar type 2 cell lineage^5^ highlights its underexplored function in distal alveolar homeostasis.

## Supplementary Material

aamag320_Supplementary_Data
