# Supplementary material for: From idiopathic pulmonary hemosiderosis to ENaC channelopathy: a novel SCNN1G mutation
Source: Am J Respir Crit Care Med. 2026 Jul 7;212(10):2628–31. doi: 10.1093/ajrccm/aamag320 (PMC13624788; doi:10.1093/ajrccm/aamag320)
Supplement: Supplementary file 1 [file aamag320_supplementary_data.zip › Supplementary Table S1.docx]

| Supplementary Table S1. Comprehensive clinical, biochemical, and immunological evaluation for differential diagnosis. | | | |
| --- | --- | --- | --- |
| **Category** | **Parameter / Test Item** | **Patient Result** | **Reference Range** |
| Hemodynamics & Electrolytes | Systolic Blood Pressure | 105 | 90–120 mmHg |
|  | Diastolic Blood Pressure | 79 | 60–80 mmHg |
|  | Serum Potassium (K+) | 4.0 | 3.5–5.3 mmol/L |
|  | Serum Sodium (Na+) | 141 | 137–147 mmol/L |
|  | Serum Chloride (Cl-) | 108 | 99–110 mmol/L |
| Endocrine (RAAS Profile) | Renin Activity | 1.05 | Supine: 0.15–2.33 ng/ml/hr Upright: 0.10–6.56 ng/ml/hr |
|  | Angiotensin II | 51.9 | Supine: 25–60 pg/ml  Upright: 50–120 pg/ml |
|  | Aldosterone | 61.9 | Supine: 30–160 pg/ml  Upright: 70–300 pg/ml |
| Connective Tissue Disease | Antinuclear antibodies (ANA) IgG | Negative (-) | Negative |
|  | Anti-dsDNA antibodies | Negative (-) | Negative |
|  | Extractable Nuclear Antigen (ENA) panel* | Negative (-) | Negative |
| Vasculitis & Others | p-ANCA | Negative (-) | Negative |
|  | c-ANCA | Negative (-) | Negative |
|  | Myeloperoxidase (MPO) IgG | 2.8 | ≤ 20 AU/mL |
|  | Proteinase 3 (PR3) IgG | <2.0 | ≤ 20 AU/mL |
| Coagulopathy | Prothrombin time (PT) | 14.3 | 11–16 s |
|  | Activated partial thromboplastin time (APTT) | 42.8 | 25–45 s |
|  | International normalized ratio (INR) | 1.19 | 0.73–1.28 |
|  |  |  |  |
| *Note: *The 17-item ENA panel included antibodies against: nucleosomes, histones, SmD1, PCNA, RPP/Po, SSA/Ro60, SSA/Ro52, SSB/La, CenpB, Scl-70, U1-snRNP, AMA-M2, Jo-1, PM-Scl, Mi-2, and Ku.* | | | |
|  |  |  |  |
|  |  |  |  |
|  |  |  |  |
